# Clinical Profile of Documented Diabetic Peripheral Neuropathy by GLP-1-Based Medication Status: A Cross-Sectional Study

**DOI:** 10.3390/medicina62091816

**Published:** 2026-09-21

**Authors:** Anca Cristina Ferician, Timea Claudia Ghitea, Mihaela Simona Popoviciu

**Affiliations:** 1 Department of Medical Disciplines, Faculty of Medicine and Pharmacy, University of Oradea, 410068 Oradea, Romania; anca.ferician@uoradea.ro; 2 Pharmacy Department, Faculty of Medicine and Pharmacy, University of Oradea, 410087 Oradea, Romania; 3 Department of Preclinical Disciplines, Faculty of Medicine and Pharmacy, University of Oradea, 410028 Oradea, Romania; mihaela.popoviciu@didactic.uoradea.ro

**Keywords:** documented diabetic peripheral neuropathy, documented GLP-1-based therapy, multimorbidity, heart failure, lower-limb amputation, prevalence ratio, exploratory cross-sectional study, routinely collected health data

## Abstract

*Background and Objectives*: This exploratory single-center retrospective cross-sectional study characterized patients with documented diabetic peripheral neuropathy (DPN) according to documented GLP-1-based medication status. GLP-1-based medication status was defined by a hospital medication-field entry for exenatide, dulaglutide, semaglutide, a fixed-ratio insulin/GLP-1 receptor agonist combination, or tirzepatide, a dual GIP/GLP-1 receptor agonist. *Materials and Methods*: Medication-level records from a hospital-defined February 2024–February 2026 reporting window were aggregated by PatID for 4120 unique patients in active-patient exports from the Clinical County Emergency Hospital Bihor. The files lacked patient-level encounter, diagnosis, and treatment dates; therefore, they were analyzed as an undated reporting snapshot. DPN and diabetes type were defined from export fields and were not independently clinically validated. *Results*: GLP-1-based medications were documented in 852 patients (20.7%). Patients with these medications in their records had longer documented diabetes duration (14.85 ± 8.64 vs. 11.72 ± 8.73 years; *p* < 0.001) and a higher documented comorbidity count (2.64 ± 1.65 vs. 1.89 ± 1.55; *p* < 0.001). Heart failure was documented in 35.7% versus 22.2%, and any lower-limb amputation in 3.3% versus 1.2%. For heart failure, the adjusted odds ratio was 1.68 (95% CI 1.41–1.99) in the primary model and 1.37 (1.12–1.67) in the expanded exploratory model; the corresponding robust Poisson prevalence ratio was 1.45 (1.30–1.63). Because only 66 amputations were recorded, amputation was retained as a descriptive, unadjusted outcome and was not modeled multivariably. *Conclusions*: A GLP-1-based medication record identified a clinically complex subgroup with documented DPN. The findings are exploratory and hypothesis-generating; because treatment and outcome dates were unavailable, they do not establish temporality, treatment benefit, or treatment harm.

## 1. Introduction

Diabetic peripheral neuropathy (DPN) is among the most common chronic complications of diabetes and contributes to pain, sensory loss, functional impairment, foot ulceration, infection, and lower-limb amputation. Its clinical burden extends beyond neurological manifestations because neuropathy frequently coexists with peripheral vascular disease, renal dysfunction, obesity, and cardiovascular disease. Lower-limb amputation therefore often reflects the combined effects of neuropathy, vascular impairment, infection, impaired wound healing, and long-standing metabolic disease rather than an isolated neurological complication [1,2,3,4,5].

Current diabetes guidelines recommend selecting glucose-lowering therapy according to cardiovascular, kidney, heart-failure, glycemic, and weight-related priorities. GLP-1 receptor agonists and related incretin-based therapies are consequently documented more often in many patients with type 2 diabetes, obesity, established cardiovascular disease, or a need for treatment modification. Patients with these medications in their records may therefore differ systematically from those without such records before treatment selection [6].

The relationship between GLP-1 receptor agonists and DPN remains incompletely defined. Experimental and early clinical evidence suggests possible neuroprotective, anti-inflammatory, vascular, and metabolic effects, but clinical evidence is limited and has not established a definitive effect on peripheral neuropathy. Likewise, available meta-analytic evidence on lower-limb outcomes is not directly applicable to all patients with established DPN [7,8,9].

Real-world data may help characterize patients with documented DPN according to documented GLP-1-based medication status in routine practice. However, cross-sectional comparisons are particularly vulnerable to confounding by indication: longer diabetes duration, obesity, cardiovascular or kidney disease, previous treatment failure, and greater overall complication burden may simultaneously influence treatment selection and the prevalence of adverse clinical conditions. Accordingly, positive associations between a GLP-1-based medication record and prevalent conditions should not be interpreted as causal treatment effects [10,11,12,13,14,15,16].

This exploratory study aimed to characterize the demographic, metabolic, therapeutic, and documented multimorbidity profile of patients with documented DPN according to documented GLP-1-based medication status. We additionally estimated descriptive cross-sectional associations with prevalent heart failure. Lower-limb amputation was evaluated descriptively because of the limited event count. The estimand was the association between therapy status recorded in the common reporting snapshot and the prevalence of the corresponding condition; no causal treatment effect or temporal ordering was assumed. Because GLP-1 use was concentrated in type 2 diabetes, type 2 diabetes-only analyses were conducted as clinically motivated exploratory sensitivity analyses for heart failure and the composite outcome, while the all-diabetes cohort was retained for descriptive completeness.

## 2. Materials and Methods

### 2.1. Design and Data Sources

This exploratory single-center retrospective cross-sectional analysis used active-patient exports from the Clinical County Emergency Hospital Bihor, Oradea, Romania. February 2024 through February 2026 was the hospital-defined reporting window used to generate the source exports; it was not a patient-level follow-up period. Data were accessed and extracted between 15 and 30 July 2026, after ethics approval had been granted on 13 July 2026 (approval No. 22733). The files did not contain patient-level extraction timestamps, encounter dates, treatment start or stop dates, or diagnosis- or complication-onset dates. They also did not indicate whether an entry represented a medication or diagnosis active at extraction, the most recent available record, or an accumulated historical entry. Accordingly, the linked exports were analyzed as an undated cross-sectional reporting snapshot rather than as observations from a dated clinical visit or a longitudinal interval. In this database, “active” denotes inclusion in the corresponding hospital export at extraction; it does not establish a new diagnosis, a current prescription, or temporal precedence. Restriction to active patients may exclude inactive, transferred, deceased, or lost-to-follow-up patients and may introduce selection bias. The supplied files did not preserve the underlying ICD-code algorithms or a separate clinician-adjudication field, so diagnoses were operationalized as documented in the relevant export. Reporting was guided by the STROBE and RECORD recommendations [17,18]. The completed STROBE and RECORD checklists are provided as Supplementary Files S2 and S3, respectively.

The documented-DPN export contained 7660 medication-level rows representing 4120 unique PatID values. Separate exports represented heart failure, above- and below-ankle amputation, stroke, ischemic heart disease, myocardial infarction/CABG/angioplasty, hypertension, nephropathy, dyslipidemia, blindness, acute ulcer/gangrene, claudication, angina, and postural hypotension. The exports did not distinguish inpatient from outpatient encounters and did not provide the number of consultations per patient.

### 2.2. Cohort Construction and Linkage

Eligibility was defined by presence in the hospital export filtered as “Complicatii: Neuropatie periferica” and a valid PatID. All 7660 rows had a PatID; therefore, no record was excluded for a missing identifier. No additional minimum-age or diabetes-duration threshold was imposed. Encounter-frequency criteria were not applied because visit-count data were unavailable. Documented DPN was defined operationally as membership in this export. The source files did not preserve the ICD codes or clinical criteria used to generate the export, did not identify whether the diagnosis followed a neurology or endocrinology assessment, and did not document confirmation by neurological examination, monofilament testing, vibration testing, or nerve-conduction studies. Thus, DPN was defined via data extraction and should not be interpreted as independently clinically confirmed DPN. Repeated medication rows were collapsed to one patient-level record by PatID; no duplicate patient identifiers remained after aggregation. Binary condition variables were created by membership of each PatID in the corresponding export. Heart failure and amputation were defined by membership in the respective hospital exports; any amputation was the union of the below-ankle and above-ankle exports. The data did not permit independent adjudication of alternative neuropathy causes, including alcohol exposure, vitamin B12 deficiency, hypothyroidism, autoimmune disease, advanced renal disease, or neurotoxic-drug exposure. Diagnostic misclassification and selection bias related to the active-patient sampling frame are therefore possible.

### 2.3. Medication Classification

Documented GLP-1-based medication status was defined as the presence of at least one matching entry in the hospital medication field at the reporting snapshot. It indicates that a medication appeared in the available hospital record, not that it was dispensed, current, taken as prescribed, or used continuously. Prescription and expiration dates, dispensing records, dose, treatment duration, and adherence measures were unavailable. Medications prescribed outside the hospital may not have been captured. Brand names and repeated prescriptions were mapped to active ingredients and collapsed at the patient and medication-subclass levels; subclass categories were not mutually exclusive when a patient had more than one recorded product. The GLP-1-based group included exenatide (Bydureon/Byetta), dulaglutide (Trulicity), semaglutide (Ozempic/Rybelsus), fixed-ratio insulin/GLP-1 receptor agonist combinations (Xultophy/Suliqua), and tirzepatide (Mounjaro). Tirzepatide was retained as a separate dual GIP/GLP-1 subclass. Patient-level subclass counts were exenatide 483, dulaglutide 142, semaglutide 127, fixed-ratio insulin/GLP-1 combinations 134, and tirzepatide 1.

The medication dictionary identified 1223 patients with a sulfonylurea record, including 7 with Minidiab (glipizide), and 347 with a DPP-4 inhibitor record, including records for Dalmevin (vildagliptin). One ambiguous entry, DALTESSA 80/850, was not classified as a DPP-4 inhibitor. A classifiable glucose-lowering medication could not be assigned to 106 patients because the medication field was empty, contained a placeholder, or listed only neuropathy-supportive or testing products. Because treatment indication was unavailable, the 53 patients with type 1 diabetes and 2 patients with other diabetes types who had a GLP-1-based medication record cannot be classified as on-label or off-label users. The complete brand-name-to-active-ingredient mapping is provided in Supplementary Table S1.

### 2.4. Variables, Missing Data, and Data-Quality Checks

Age, sex, diabetes duration, laboratory measurements, anthropometric variables, and diabetes type were extracted from the documented-DPN export. The presence of diabetes was implicit in the source export, and diabetes type (type 1, type 2, or other) was taken from its administrative diabetes-type field. The supplied data did not retain the ICD codes, physician-recorded diagnostic text, laboratory criteria, medication-based rules, or other source logic used to assign diabetes presence or type; these classifications therefore could not be independently validated and are reported as documented/data-extraction-defined categories. The documented comorbidity count was the unweighted sum of 13 binary conditions: heart failure, any lower-limb amputation, hypertension, ischemic heart disease, myocardial infarction/CABG/angioplasty, stroke, nephropathy, dyslipidemia, blindness, acute ulcer/gangrene, claudication, angina, and postural hypotension. Each condition contributed one point. The heart-failure/amputation composite and the separate above- and below-ankle categories were excluded from this count to avoid double counting. Clinically overlapping diagnostic categories, such as ischemic heart disease and myocardial infarction/CABG/angioplasty, remained separate components. The observed age range was 17–96 years; no additional minimum-age threshold was imposed. HbA1c, estimated glomerular filtration rate, urinary albumin-to-creatinine ratio, treatment start dates, treatment duration, dose, adherence, ulcer severity, laterality, and peripheral arterial disease staging were unavailable. Analyses used available observations without imputation. Prespecified data-plausibility thresholds were height outside 120–220 cm and BMI >80 kg/m^2^. Weight and BMI were available in 282 and 273 patients, respectively; after exclusion of two BMI values > 80 kg/m^2^, 271 observations remained. Height was recorded much more frequently than weight (2831 vs. 282 patients); the available files did not establish whether this discrepancy arose from the clinical data structure, data entry, or data transfer.

### 2.5. Statistical Analysis

Continuous variables are summarized as the mean ± SD in the primary table and were compared with the Mann–Whitney U test; these *p* values test differences in distributions/ranks and not differences in means. Continuous-variable distributions were assessed using histograms and Q–Q plots. Categorical variables were compared using Pearson’s chi-square test or Fisher’s exact test for sparse tables. All tests were two-tailed, and the nominal significance level was α = 0.05. Because numerous comorbidities and medication classes were compared, the *p* values in Table 1, Table 2 and Table 3 are explicitly exploratory; no multiplicity adjustment was applied. Logistic regression used complete cases and reported odds ratios with 95% confidence intervals. Modified Poisson models with a log link and robust covariance estimation were fitted as exploratory sensitivity analyses to estimate adjusted prevalence ratios for heart failure and the heart-failure/amputation composite, using the same covariates as the primary logistic models. Because only 66 amputations were recorded, multivariable amputation models were removed to avoid sparse-event overfitting; amputation is reported only as counts, prevalence, and an unadjusted between-group comparison. Statistical analyses were performed using IBM SPSS Statistics, version 30.0 (IBM Corp., Armonk, NY, USA).

For heart failure and the heart-failure/amputation composite, the primary logistic model included documented GLP-1-based medication status as the exposure variable and age, sex, diabetes duration, insulin, SGLT2 inhibitor, and metformin-containing medication records as covariates. Age, sex, and diabetes duration were selected as clinically relevant demographic and disease-duration factors; medication variables were included as markers of treatment composition and possible disease severity or treatment selection. Their timing relative to GLP-1-based medication status was unavailable. The type 2 diabetes-restricted models were clinically defined sensitivity analyses. Expanded exploratory models added diabetes type, hypertension, ischemic heart disease, myocardial infarction/CABG/angioplasty, stroke, nephropathy, and dyslipidemia. These conditions may influence treatment selection but may also lie on disease pathways or be recorded after treatment selection; consequently, the expanded models are susceptible to over-adjustment and collider bias and are not interpreted as more valid causal estimates. Robust Poisson and expanded-comorbidity models were additional exploratory sensitivity analyses. Age and diabetes duration were entered as continuous variables. Analyses used complete cases, and the effective sample size for each model is reported in Table 4. Formal results for linearity in the logit, multicollinearity, model fit and calibration, and influential observations were unavailable and are not reported. No multivariable model was retained for amputation, and the combined outcome was interpreted as a descriptive indicator largely driven by heart failure.

### 2.6. Ethics

The study was approved by the Ethical Committee of the Clinical County Emergency Hospital Bihor, Oradea, Romania (approval No. 22733, 13 July 2026). The approval date followed the February 2024–February 2026 clinical data window, consistent with the retrospective design. Data access and extraction for this study took place between 15 and 30 July 2026, after ethics approval. General informed consent was obtained from all subjects involved in the study. Written informed consent has been obtained from the patient(s) to publish this paper.

## 3. Results

### 3.1. Cohort Derivation and Clinical Characteristics

**Figure 1:**
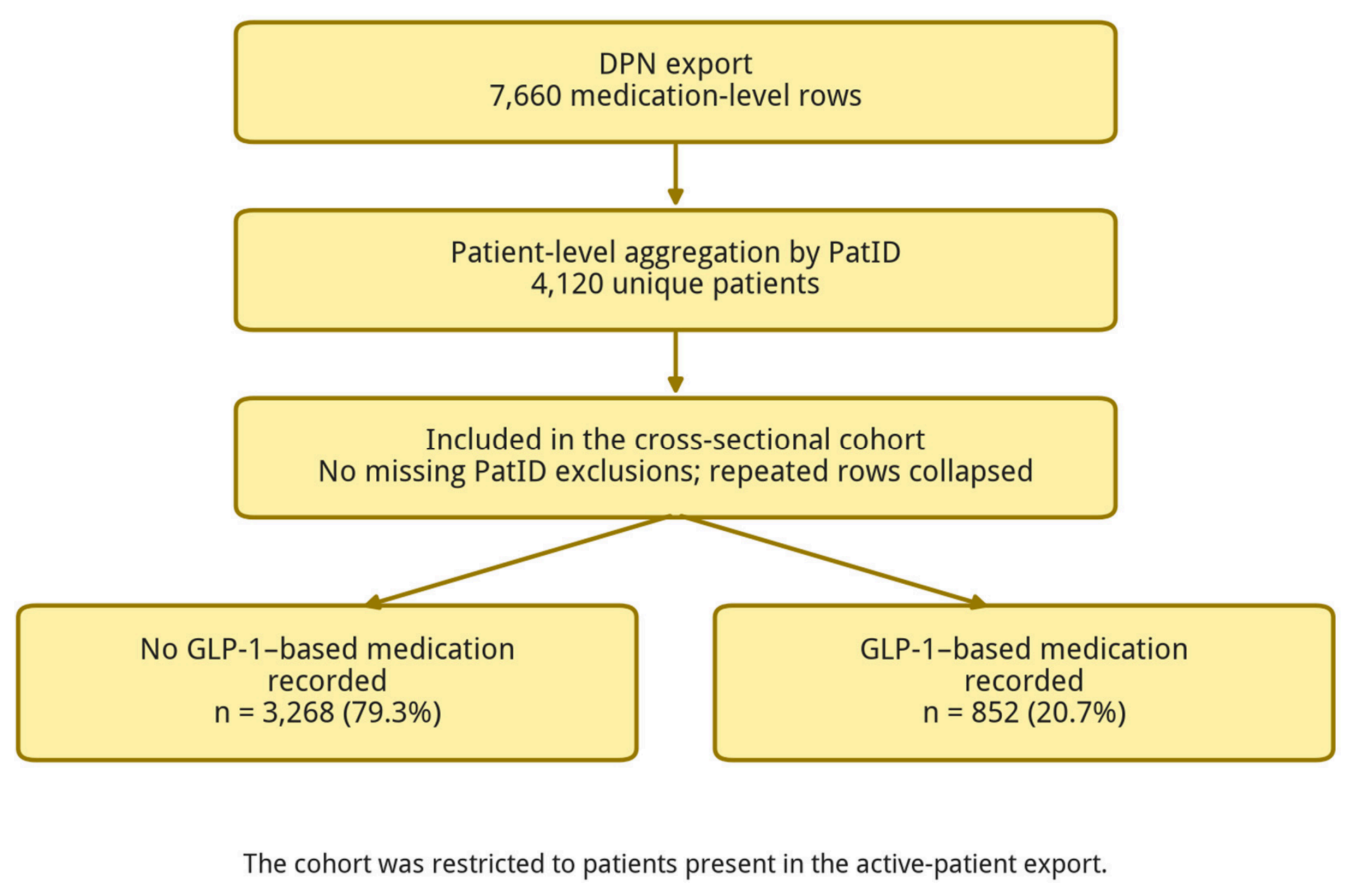
Patient flow from medication-level documented-DPN export rows to the patient-level groups defined by documented GLP-1-based medication status. DPN, documented diabetic peripheral neuropathy in this cohort; PatID, patient identifier.

The participant-selection sequence was 7660 medication-level documented-DPN rows, followed by 4120 unique PatID values after patient-level aggregation, of whom 852 had a documented GLP-1-based medication and 3268 did not. The cohort included 3769 patients with documented type 2 diabetes, 346 with documented type 1 diabetes, and 5 with other documented diabetes types. All 4120 records had a PatID, and repeated medication rows were collapsed rather than treated as separate patients. The analysis therefore describes an active-export-defined clinical cohort rather than a prospectively recruited or population-based sample (Figure 1).

The between-group age comparison was not statistically significant (65.90 ± 10.31 vs. 65.16 ± 11.35 years; *p* = 0.118). The proportion of women was modestly lower among patients with a GLP-1-based medication record (54.5% vs. 59.3%; *p* = 0.011), whereas type 2 diabetes was slightly more frequent (93.5% vs. 90.9%; overall diabetes-type comparison *p* = 0.021). The clearest clinical difference was diabetes duration: the documented GLP-1-based medication group had a mean duration of 14.85 years compared with 11.72 years in the group without such a record (*p* < 0.001).

Among the limited available laboratory measurements, between-group comparisons of fasting glucose and creatinine were not statistically significant. Patients with a documented GLP-1-based medication had lower recorded total cholesterol and higher recorded triglycerides, body weight, and BMI in the available-case samples. After plausibility exclusions, BMI was included in the comparison for 68/852 patients with a GLP-1-based medication record (8.0%) and 203/3268 patients without such a record (6.2%), weight (8.6% vs. 6.4%), creatinine (12.2% vs. 11.4%), and triglycerides (9.3% vs. 8.7%), while cholesterol availability was identical (9.5% vs. 9.5%). Given the very low availability of anthropometric and lipid measurements, these comparisons are descriptive and exploratory and cannot be assumed to represent the full cohort.

Table 1 uses one primary summary measure (mean ± SD) for continuous variables; observation counts are retained where data were incomplete.

### 3.2. Treatment Patterns

Insulin records were similar in the two groups (47.2% vs. 45.1%; exploratory *p* = 0.285). SGLT2 inhibitor records were more frequent among patients with a documented GLP-1-based medication (28.4% vs. 15.6%; exploratory *p* < 0.001), whereas metformin-containing, sulfonylurea, and DPP-4 inhibitor records were less frequent (all exploratory *p* < 0.001). These comparisons describe medication records and not verified medication use.

When the GLP-1 component itself was counted, the documented GLP-1-based medication group had more recorded glucose-lowering classes than the comparison group (mean 2.42 ± 0.89 vs. 1.64 ± 0.77; exploratory *p* < 0.001). After the GLP-1 component was excluded, the group had slightly fewer additional classes (1.42 ± 0.89 vs. 1.64 ± 0.77; exploratory *p* < 0.001). The findings therefore indicate a different treatment composition rather than uniformly greater treatment intensity.

### 3.3. Multimorbidity and Cardio-Limb Outcomes

The mean unweighted number of documented comorbid conditions was higher in the documented GLP-1-based medication group (2.64 ± 1.65) than in the group without such a record (1.89 ± 1.55; exploratory *p* < 0.001). The largest absolute differences involved dyslipidemia, hypertension, ischemic heart disease, and heart failure. These were prevalent conditions documented in the reporting snapshot and should not be interpreted as incident complications or baseline disease states.

Linkage identified 1028 patients with prevalent heart failure and 66 with any documented lower-limb amputation. Of the latter, 53 appeared in the below-ankle export and 19 in the above-ankle export; 6 patients occurred in both, explaining why the combined count was 66 rather than 72. Any amputation was documented in 28 patients with a GLP-1-based medication record (3.3%) and 38 patients without such a record (1.2%; *p* < 0.001). The heart-failure/amputation composite was documented in 318 (37.3%) and 749 (22.9%) patients, respectively (*p* < 0.001).

Blindness was rare but more frequently documented in the GLP-1-based medication group (1.2% vs. 0.4%; exploratory Fisher *p* = 0.019). Acute ulcer or gangrene was recorded in 18 patients (0.8% vs. 0.3%; exploratory Fisher *p* = 0.075). Claudication, angina, and postural hypotension were represented by only one, one, and two patients, respectively. These very small counts may reflect incomplete ascertainment, but the available data cannot establish the cause; comparative inference is not meaningful for these outcomes.

### 3.4. Adjusted and Sensitivity Analyses

In unadjusted analyses, a documented GLP-1-based medication was associated with prevalent heart failure (OR 1.95; PR 1.61) and the heart-failure/amputation composite (OR 2.00; PR 1.63). In the primary models adjusted for age, sex, diabetes duration, insulin, SGLT2 inhibitor, and metformin-containing medication records, the corresponding adjusted ORs were 1.68 (95% CI 1.41–1.99) and 1.71 (1.44–2.03). Robust Poisson sensitivity estimates were 1.45 (1.30–1.63) and 1.46 (1.31–1.63), respectively. Odds ratios and prevalence ratios answer different descriptive questions, particularly because heart failure was common. Any amputation was documented in 3.3% versus 1.2% (unadjusted *p* < 0.001); no adjusted amputation estimate is reported.

Adding diabetes type produced modest attenuation: the adjusted OR was 1.62 (95% CI 1.36–1.92) for heart failure and 1.65 (1.39–1.96) for the composite. Results were directionally consistent in the 3768 complete observations with documented type 2 diabetes: 1.58 (1.32–1.89) for heart failure and 1.61 (1.35–1.93) for the composite. These type 2 diabetes-only estimates are presented as sensitivity analyses because the indication and timing of GLP-1 use were unavailable.

The expanded exploratory model added documented diabetes type, hypertension, ischemic heart disease, myocardial infarction/CABG/angioplasty, stroke, nephropathy, and dyslipidemia. The association with prevalent heart failure was 1.37 (95% CI 1.12–1.67; *p* = 0.002), and the composite estimate was 1.41 (1.15–1.72; *p* < 0.001). The attenuation is compatible with differences in documented multimorbidity, but it cannot identify confounding by true pre-exposure factors because variable timing was unavailable. Some added conditions may be determinants of treatment selection, intermediates, or consequences of shared disease processes; over-adjustment and collider bias are therefore possible. No adjusted amputation model was retained because only 66 events were recorded.

### 3.5. Data Completeness and Robustness Checks

Repeated medication rows did not contain conflicting values for the demographic or clinical fields used in the analysis. Nevertheless, laboratory and anthropometric measurements were highly incomplete: cholesterol, triglycerides, creatinine, weight, and BMI were each available for fewer than 12% of the cohort. Group-specific availability is shown in Table 5. No imputation was performed, denominators are reported explicitly, and the available cases cannot be assumed to represent the entire cohort.

The two BMI values above 80 kg/m^2^ were 81.36 and 346.94 kg/m^2^ and were linked to recorded heights of 104 and 70 cm. They were not manually corrected because the intended heights could not be inferred. Excluding both observations yielded 271 evaluable patients and preserved the direction of the available-case comparison; however, removal of two implausible values does not address selection bias arising from the very limited availability of BMI measurements. The BMI analysis is therefore descriptive and exploratory and should not be used to define the anthropometric profile of the full cohort. The large difference between height and weight availability could reflect the clinical data structure, data entry, or data transfer, but the supplied exports do not distinguish among these explanations.

## 4. Discussion

This exploratory analysis identified 4120 active-export patients with documented DPN, including 852 with a documented GLP-1-based medication. This group had longer documented diabetes duration, a different glucose-lowering medication composition, and a greater burden of documented prevalent multimorbidity. These findings describe clinical complexity and treatment selection in an undated hospital reporting snapshot; they do not demonstrate that a medication caused or prevented any condition.

The association with prevalent heart failure changed from aOR 1.68 in the primary model to aOR 1.37 in the expanded exploratory model, while the robust Poisson sensitivity estimate was PR 1.45. Because the added cardiovascular, renal, and metabolic conditions may have been recorded before, after, or contemporaneously with medication status, the attenuation should not be interpreted as proof of confounding control. Inclusion of possible intermediates or common effects may introduce over-adjustment or collider bias [10,11].

Patients with a documented GLP-1-based medication had longer recorded diabetes duration than those without such a record. This supports greater documented clinical complexity but does not establish a more advanced disease stage. Neuropathy severity, longitudinal glycemic control, albuminuria, eGFR, HbA1c, and other markers required to support that interpretation were not assessed [1,2].

Body weight and BMI were higher in the documented GLP-1-based medication group within the small available-case sample. BMI was available for 8.0% of this group and 6.2% of the comparison group, with 93.4% missing overall. These anthropometric comparisons are descriptive, exploratory, and highly selected; mechanistic interpretations and generalization to the full cohort are not warranted [19,20,21].

The medication pattern indicates a different composition of documented glucose-lowering treatment records: SGLT2 inhibitor records were more frequent and sulfonylurea, DPP-4 inhibitor, and metformin-containing records were less frequent in the documented GLP-1-based medication group. After the GLP-1 component was excluded, this group did not have more additional glucose-lowering classes. This is a direct description of the extracted medication fields and should not be interpreted as verified treatment intensity, dispensing, adherence, or duration [22,23,24,25].

Heart failure was more prevalent among patients with a documented GLP-1-based medication, but the cross-sectional design and the common outcome make the OR less intuitive than a prevalence ratio; both measures are reported [12,13]. The association may reflect clinical selection and co-occurring documented conditions. Treatment start dates, disease-onset dates, HbA1c, eGFR, and albuminuria were unavailable, so no causal interpretation is justified.

The relationship between incretin-based therapies and DPN remains uncertain. The present analysis is a real-world phenotyping study of documented therapy status, not an evaluation of neuropathy response. The exposure combined conventional GLP-1 receptor agonists, fixed-ratio insulin/GLP-1 combinations, and tirzepatide, which differ in molecular targets and formulation; subclass counts are reported, and this heterogeneity is an additional reason to avoid a class-wide mechanistic or causal interpretation [6,14,15,16].

Lower-limb amputation was uncommon but more frequently documented in the GLP-1-based medication group (28/852, 3.3%) than in the comparison group (38/3268, 1.2%; unadjusted *p* < 0.001). The full cohort contained only 66 amputation events, including 54 among patients with documented type 2 diabetes. Therefore, no multivariable amputation model is retained. The descriptive difference is hypothesis-generating, may be affected by confounding by indication and diagnostic misclassification, and should not be interpreted as evidence of treatment harm.

External longitudinal studies may report different limb-outcome patterns, but they are not directly comparable with this undated, cross-sectional, export-defined analysis. The present data cannot determine whether therapy preceded amputation, whether treatment indication differed by limb risk, or whether adherence and duration varied between groups.

The adjusted analyses describe cross-sectional associations between documented medication status and prevalent heart failure and the heart-failure/amputation composite. The composite added a broad descriptive indicator of cardio-limb burden but was dominated by heart-failure events and did not provide an independent confirmatory endpoint. The expanded models were treated as exploratory sensitivity analyses, not as a causal adjustment sequence. Amputation was retained only as an unadjusted descriptive outcome [26,27,28,29,30].

Only 54 amputations occurred among patients with documented type 2 diabetes. The exports also lacked amputation date, laterality, ulcer-history completeness, peripheral arterial disease ascertainment, treatment start date, dose, and adherence. These constraints precluded a defensible adjusted amputation analysis; the revised manuscript therefore reports counts, prevalence, and the unadjusted between-group comparison only. This result is strictly exploratory and cannot estimate incident limb risk or a treatment effect.

The medication dictionary mapped commercial names to active ingredients and included Minidiab as glipizide and Dalmevin as vildagliptin. The resulting patient-level counts were 1223 for sulfonylureas and 347 for DPP-4 inhibitors. The availability of the mapping dictionary and analysis code is described in the Data Availability Statement.

Anthropometric analyses remain highly selected because BMI was available in only 6.6% of patients overall, with modestly different availability by therapy group. The two implausible BMI values were excluded in a sensitivity analysis, but this does not resolve selection bias. These findings cannot establish the anthropometric profile of the full cohort.

Strengths include patient-level aggregation, reproducible linkage across the supplied exports, explicit medication mapping, type 2 diabetes-only and robust prevalence-ratio sensitivity analyses for heart failure and the composite outcome, and group-specific reporting of missingness. Limitations include export-defined DPN and diabetes type without preserved ICD codes, physician diagnostic text, laboratory criteria, specialist adjudication, or confirmatory neurological testing; restriction to active patients; an administrative reporting window that did not provide patient-level index dates or distinguish current, most recent, and historical entries; lack of treatment and outcome dates; inability to distinguish care setting; heterogeneous GLP-1-based medication records; inclusion of a small documented type 1 diabetes subgroup; severe missingness for metabolic and anthropometric variables; sparse limb events; incomplete peripheral arterial disease and ulcer ascertainment; and possible diagnostic misclassification, residual confounding, over-adjustment, and collider bias.

## 5. Conclusions

A documented GLP-1-based medication identified an exploratory subgroup of patients with documented DPN characterized by longer recorded diabetes duration, a different documented medication composition, and greater documented prevalent multimorbidity. Associations with documented heart failure were exploratory and non-causal; the amputation comparison was strictly descriptive and unadjusted because only 66 events were recorded. Because the exports lacked verified treatment and outcome dates and did not distinguish current, most recent, and accumulated historical entries, the findings do not establish temporality, treatment benefit, treatment harm, or incident risk.

## Figures and Tables

**Table 1:** Demographic, clinical, laboratory, and anthropometric characteristics of patients with documented diabetic peripheral neuropathy, stratified by documented GLP-1-based medication status.

Variable	Total	No GLP-1	GLP-1	*p* †
Age, years	65.31 ± 11.14; *n* = 4120	65.16 ± 11.35; *n* = 3268	65.90 ± 10.31; *n* = 852	0.118
Female sex	2401 (58.3%)	1937 (59.3%)	464 (54.5%)	0.011
Diabetes duration, years	12.37 ± 8.80; *n* = 4119	11.72 ± 8.73; *n* = 3267	14.85 ± 8.64; *n* = 852	<0.001
Type 1/type 2/other diabetes	346/3769/5	293/2972/3	53/797/2	0.021
Fasting glucose, mg/dL	147.56 ± 46.85; *n* = 2270	147.35 ± 47.28; *n* = 1736	148.22 ± 45.46; *n* = 534	0.292
Total cholesterol, mg/dL	180.89 ± 53.91; *n* = 390	184.65 ± 54.68; *n* = 309	166.56 ± 48.58; *n* = 81	0.015
Triglycerides, mg/dL	158.66 ± 114.44; *n* = 363	153.97 ± 117.36; *n* = 284	175.50 ± 102.21; *n* = 79	0.014
Creatinine, mg/dL	1.07 ± 0.73; *n* = 477	1.06 ± 0.70; *n* = 373	1.10 ± 0.83; *n* = 104	0.788
Body weight, kg	87.54 ± 20.35; *n* = 282	84.91 ± 20.07; *n* = 209	95.07 ± 19.35; *n* = 73	<0.001
BMI, kg/m^2^	31.52 ± 6.20; *n* = 271	30.45 ± 5.95; *n* = 203	34.72 ± 5.90; *n* = 68	<0.001
Documented comorbidity count	2.05 ± 1.60; *n* = 4120	1.89 ± 1.55; *n* = 3268	2.64 ± 1.65; *n* = 852	<0.001

Continuous values are the mean ± SD; *n*. *p* values for continuous variables are from the Mann–Whitney U test and refer to differences in distributions/ranks, not differences in means. Laboratory and anthropometric results are available-case analyses. The BMI row excludes two values > 80 kg/m^2^ generated by implausible heights. † *p* values are exploratory, and no multiplicity adjustment was applied.

**Table 2:** Documented glucose-lowering medication classes among patients with documented diabetic peripheral neuropathy, overall and according to documented GLP-1-based medication status.

Treatment	Total	No GLP-1	GLP-1	*p* †
Insulin	1877 (45.6%)	1475 (45.1%)	402 (47.2%)	0.285
SGLT2 inhibitor	751 (18.2%)	509 (15.6%)	242 (28.4%)	<0.001
Metformin-containing	2286 (55.5%)	1877 (57.4%)	409 (48.0%)	<0.001
Sulfonylurea	1223 (29.7%)	1105 (33.8%)	118 (13.8%)	<0.001
DPP-4 inhibitor	347 (8.4%)	316 (9.7%)	31 (3.6%)	<0.001
Acarbose	34 (0.8%)	34 (1.0%)	0 (0.0%)	<0.001
Meglitinide	17 (0.4%)	14 (0.4%)	3 (0.4%)	1.000
Thiazolidinedione	20 (0.5%)	19 (0.6%)	1 (0.1%)	0.098
Exenatide (Bydureon/Byetta)	483 (11.7%)	0	483 (56.7%)	—
Dulaglutide (Trulicity)	142 (3.4%)	0	142 (16.7%)	—
Semaglutide (Ozempic/Rybelsus)	127 (3.1%)	0	127 (14.9%)	—
Fixed-ratio insulin/GLP-1 (Xultophy/Suliqua)	134 (3.3%)	0	134 (15.7%)	—
Tirzepatide (Mounjaro)	1 (0.02%)	0	1 (0.12%)	—

Data are patient-level *n* (%). A classifiable glucose-lowering medication could not be assigned to 106 patients (2.6%) because the medication field was empty, contained a placeholder, or listed only neuropathy-supportive or testing products. Subclass rows are not mutually exclusive. Brand names were mapped using the medication dictionary described in Section 2.3. † *p* values are exploratory, and no multiplicity adjustment was applied.

**Table 3:** Prevalence of documented cardiovascular, renal, and diabetes-related conditions among patients with documented diabetic peripheral neuropathy, overall and according to documented GLP-1-based medication status.

Condition	Total	No GLP-1	GLP-1	*p* †
Heart failure	1028 (25.0%)	724 (22.2%)	304 (35.7%)	<0.001
Heart failure and/or amputation	1067 (25.9%)	749 (22.9%)	318 (37.3%)	<0.001
Any lower-limb amputation	66 (1.6%)	38 (1.2%)	28 (3.3%)	<0.001
Below-ankle amputation	53 (1.3%)	33 (1.0%)	20 (2.3%)	0.002
Above-ankle amputation	19 (0.5%)	8 (0.2%)	11 (1.3%)	<0.001
Hypertension	2847 (69.1%)	2167 (66.3%)	680 (79.8%)	<0.001
Ischemic heart disease	1669 (40.5%)	1239 (37.9%)	430 (50.5%)	<0.001
MI/CABG/angioplasty	206 (5.0%)	147 (4.5%)	59 (6.9%)	0.004
Stroke	283 (6.9%)	196 (6.0%)	87 (10.2%)	<0.001
Nephropathy	824 (20.0%)	588 (18.0%)	236 (27.7%)	<0.001
Dyslipidemia	1473 (35.8%)	1064 (32.6%)	409 (48.0%)	<0.001
Blindness	24 (0.6%)	14 (0.4%)	10 (1.2%)	0.019
Acute ulcer/gangrene	18 (0.4%)	11 (0.3%)	7 (0.8%)	0.075
Claudication	1 (0.02%)	0	1 (0.12%)	0.207
Angina	1 (0.02%)	1 (0.03%)	0	1.000
Postural hypotension	2 (0.05%)	2 (0.06%)	0	1.000

Data are *n* (%). Conditions are not mutually exclusive. The “heart failure and/or amputation” composite includes documented heart failure, any lower-limb amputation, or both; because heart failure events substantially outnumbered amputation events, the composite was used only as a descriptive summary of cardio-limb burden and was interpreted primarily in light of heart failure. “Any lower-limb amputation” includes below-ankle and/or above-ankle amputation, so the anatomical categories may overlap. † *p* values are unadjusted and exploratory; no multiplicity adjustment was applied. CABG, coronary artery bypass grafting; GLP-1, glucagon-like peptide-1; MI, myocardial infarction.

**Table 4:** Exploratory cross-sectional associations between documented GLP-1-based medication status and prevalent heart failure and the heart-failure/amputation composite: logistic and robust Poisson sensitivity analyses.

Outcome and Model	Events/*n*	OR or PR	95% CI	*p*
HF and/or amputation—primary model	1067/4119	1.71	1.44–2.03	<0.001
HF and/or amputation—plus diabetes type	1067/4119	1.65	1.39–1.96	<0.001
HF and/or amputation—type 2 only	1001/3768	1.61	1.35–1.93	<0.001
HF and/or amputation—expanded comorbidity model	1067/4119	1.41	1.15–1.72	<0.001
Heart failure—primary model	1028/4119	1.68	1.41–1.99	<0.001
Heart failure—plus diabetes type	1028/4119	1.62	1.36–1.92	<0.001
Heart failure—type 2 only	972/3768	1.58	1.32–1.89	<0.001
Heart failure—expanded comorbidity model	1028/4119	1.37	1.12–1.67	0.002
Heart failure—robust Poisson PR, primary covariates	1028/4119	1.45	1.30–1.63	<0.001
HF and/or amputation—robust Poisson PR, primary covariates	1067/4119	1.46	1.31–1.63	<0.001

Above-ankle amputation was not modeled because only 19 events were present. The 27 patients with both heart failure and any amputation were not treated as a separate confirmatory outcome. The combined outcome was retained only as a descriptive indicator of cardio-limb burden and was largely driven by heart failure. All model estimates concern prevalent conditions in an undated cross-sectional reporting snapshot and cannot establish temporality or causal treatment effects. Because only 66 amputations were recorded overall (54 among patients with documented type 2 diabetes), adjusted amputation models were removed, and the amputation comparison is strictly descriptive and exploratory.

**Table 5:** Data completeness, group-specific availability, and quality assessment for key variables in the study cohort.

Variable	Available *n*/N	Available, %	Result of Completeness or Quality Check	No GLP-1 *n* (%)	GLP-1 *n* (%)
Age	4120/4120	100.0	Complete	3268/3268 (100.0%)	852/852 (100.0%)
Diabetes duration	4119/4120	100.0	One missing observation	3267/3268 (100.0%)	852/852 (100.0%)
Fasting glucose	2270/4120	55.1	Available-case analysis	1736/3268 (53.1%)	534/852 (62.7%)
Total cholesterol	390/4120	9.5	Substantial missingness	309/3268 (9.5%)	81/852 (9.5%)
Triglycerides	363/4120	8.8	Substantial missingness	284/3268 (8.7%)	79/852 (9.3%)
Creatinine	477/4120	11.6	Substantial missingness	373/3268 (11.4%)	104/852 (12.2%)
Height	2831/4120	68.7	Nine values outside 120–220 cm	2249/3268 (68.8%)	582/852 (68.3%)
Weight	282/4120	6.8	Substantial missingness	209/3268 (6.4%)	73/852 (8.6%)
BMI included after plausibility exclusions	271/4120	6.6	273 values recorded; two values > 80 kg/m^2^ excluded	203/3268 (6.2%)	68/852 (8.0%)

Availability was calculated relative to the full cohort (N = 4120); group-specific availability is reported for patients without and with a documented GLP-1-based medication. Variables with missing observations were analyzed using an available-case approach. The prespecified plausibility checks were height outside 120–220 cm and BMI > 80 kg/m^2^. BMI was recorded for 273 patients before plausibility exclusions. The BMI comparison in Table 1 includes 271 patients after exclusion of two values exceeding 80 kg/m^2^. Group-specific denominators for recorded and analyzed BMI are reported separately. Percentages may differ slightly because of rounding.

## Data Availability

Patient-level hospital records cannot be publicly shared because of ethical and institutional restrictions. De-identified aggregate results are reported in the article. The medication-mapping dictionary, analysis code, and completed STROBE and RECORD checklists will be provided as Supplementary Material or through a controlled repository, subject to institutional and journal requirements.

## Supplementary Materials

The following supporting information can be downloaded at https://www.mdpi.com/article/10.3390/medicina62091816/s1. File S1. Table S1—Brand-name-to-active-ingredient mapping used for medication classification; File S2. STROBE Statement—Checklist for Cross-Sectional Studies; File S3. RECORD Statement—Completed Extension Checklist.

## Abbreviations

The following abbreviations are used in this manuscript:

Abbreviation	Definition
CI	Confidence interval
DPP-4	Dipeptidyl peptidase-4
GIP	Glucose-dependent insulinotropic polypeptide
GLP-1 RA	Glucagon-like peptide-1 receptor agonist
HF	Heart failure
OR	Odds ratio
PatID	Patient identifier
PR	Prevalence ratio
SD	Standard deviation
SGLT2	Sodium–glucose cotransporter-2
