# Supplementary material for: Clinical Profile of Documented Diabetic Peripheral Neuropathy by GLP-1-Based Medication Status: A Cross-Sectional Study
Source: Medicina (Kaunas). 2026 Sep 21;62(9):1816. doi: 10.3390/medicina62091816 (PMC13609424; doi:10.3390/medicina62091816)
Supplement: Supplementary file 1 [file medicina-62-01816-s001.zip › Supplementary Files S2.pdf]

STROBE Statement—Checklist for Cross-Sectional Studies

Manuscript: *Clinical and Multimorbidity Profile of Patients with Diabetic Peripheral Neuropathy According to Documented GLP-1–Based Medication Status: An Exploratory Single-Center Cross-Sectional Study*

Version assessed: revised manuscript, 18 pages. Page numbers refer to this version.

Item	Reporting recommendation	Location in the manuscript
1(a)	Identify the study design in the title or abstract.	Title, p. 1; Abstract, p. 2. The study is identified as an exploratory single-center retrospective cross-sectional study.
1(b)	Provide a balanced abstract summary of methods and findings.	Abstract, p. 2.
2	Explain the scientific background and rationale.	Introduction, pp. 2–3.
3	State the objectives and any prespecified hypotheses.	Introduction, p. 3. The study objective and exploratory estimand are stated; no confirmatory hypothesis is claimed.
4	Present key elements of the study design early.	Section 2.1, p. 3.
5	Describe the setting, location, and relevant dates.	Section 2.1, pp. 3–4. Clinical window: February 2024–February 2026; data access and extraction: 15–30 July 2026; hospital and location are reported.
6(a)	Give eligibility criteria and sources and methods of participant selection.	Sections 2.1–2.2, pp. 3–4; Section 3.1, p. 6; Figure 1, p. 11.
6(b)	Give matching criteria for matched studies.	Not applicable. The study was not matched.
7	Define outcomes, exposures, predictors, confounders, and effect modifiers; give diagnostic criteria where applicable.	Sections 2.1–2.5, pp. 3–6. Diagnoses were defined by membership in the corresponding hospital exports; preserved ICD codes and independent diagnostic adjudication were unavailable.
8	Give data sources and assessment methods for each variable and describe comparability between groups.	Sections 2.1–2.4, pp. 3–5. The same export-derived definitions were applied to both medication-status groups.
9	Describe efforts to address potential sources of bias.	Sections 2.1–2.5, pp. 3–6; Section 3.5, p. 12; Discussion, pp. 13–14. Selection bias, misclassification, missing-data bias, residual confounding, over-adjustment, and collider bias are discussed.
10	Explain how the study size was determined.	Sections 2.1–2.2, pp. 3–4; Section 3.1, p. 6. All 4120 unique PatID values in the eligible active-patient DPN export were included; no sampling or prospective recruitment was performed.
11	Explain how quantitative variables were handled.	Sections 2.4–2.5, pp. 5–6. Continuous variables were retained as continuous measures; summaries, comparisons, and plausibility thresholds are described.
12(a)	Describe statistical methods, including adjustment methods.	Section 2.5, pp. 5–6; Table 4, pp. 10–11. Formal results for logit linearity, multicollinearity, model fit/calibration, and influential observations are not reported in the current manuscript.
12(b)	Describe methods for subgroup and interaction analyses.	Section 2.5, pp. 5–6. Type 2 diabetes-restricted analyses are described as exploratory sensitivity analyses; no interaction analysis is reported.
12(c)	Explain how missing data were addressed.	Sections 2.4–2.5, pp. 5–6; Section 3.5 and Table 5, p. 12. Available-case and complete-case approaches were used; no imputation was performed.
12(d)	Describe analytical methods accounting for the sampling strategy, if applicable.	Sections 2.1–2.2, pp. 3–4. The cohort was an active-export-defined hospital population, not a probability sample; no survey-weighted analysis was applicable.
12(e)	Describe sensitivity analyses.	Section 2.5, pp. 5–6; Sections 3.4–3.5, pp. 10–12. Type 2 diabetes-restricted, expanded-covariate, robust Poisson, and BMI plausibility analyses are reported.
13(a)	Report numbers at each stage of the study.	Section 3.1, p. 6; Figure 1, p. 11. A total of 7660 medication-level rows were aggregated into 4120 patients, including 852 with and 3268 without a documented GLP-1-based medication.
13(b)	Give reasons for non-participation or exclusion at each stage.	Section 2.2, p. 4; Section 3.1, p. 6. No row lacked PatID; repeated medication rows were collapsed rather than excluded.
13(c)	Consider a flow diagram.	Figure 1, p. 11.
14(a)	Report participant characteristics and exposure/confounder information.	Section 3.1 and Table 1, pp. 6–7; Tables 2–3, pp. 7–9.

Item	Reporting recommendation	Location in the manuscript
14(b)	Report the number of participants with missing data for each variable.	Table 1, p. 7; Section 3.5 and Table 5, p. 12.
14(c)	Summarize follow-up time for cohort studies.	Not applicable. This was a cross-sectional analysis without longitudinal follow-up.
15	Report outcome events or summary measures.	Sections 3.2–3.4 and Tables 2–4, pp. 7–11.
16(a)	Report unadjusted and adjusted estimates with precision and identify adjustment variables.	Section 3.4 and Table 4, pp. 10–11; adjustment variables are defined in Section 2.5, pp. 5–6.
16(b)	Report category boundaries when continuous variables are categorized.	Not applicable to the regression predictors, which were entered as continuous variables. BMI and height thresholds were data-quality checks and are reported in Sections 2.4 and 3.5, pp. 5 and 12.
16(c)	Translate relative estimates into absolute risk for a meaningful period, if relevant.	Not applicable. The cross-sectional data did not establish follow-up time or incident risk.
17	Report subgroup, interaction, and sensitivity analyses.	Sections 3.4–3.5, pp. 10–12. No interaction analysis was conducted.
18	Summarize key results with reference to the objectives.	Discussion, pp. 13–14.
19	Discuss limitations, including potential bias and imprecision.	Discussion, pp. 13–14.
20	Give a cautious overall interpretation considering objectives, limitations, multiplicity, and other evidence.	Discussion, pp. 13–14; Conclusions, pp. 14–15.
21	Discuss generalizability.	Discussion, p. 14. Generalizability is limited by the single-center, active-export-defined population and incomplete clinical ascertainment.
22	State funding and the role of funders.	Funding statement, p. 15. The APC funder is identified; the funder’s role in study design, analysis, interpretation, or publication decisions is not stated in the current manuscript.

Checklist source: STROBE Statement, checklist for cross-sectional studies: https://www.strobe-statement.org/checklists/
