# Supplementary material for: Clinical Profile of Documented Diabetic Peripheral Neuropathy by GLP-1-Based Medication Status: A Cross-Sectional Study
Source: Medicina (Kaunas). 2026 Sep 21;62(9):1816. doi: 10.3390/medicina62091816 (PMC13609424; doi:10.3390/medicina62091816)
Supplement: Supplementary file 1 [file medicina-62-01816-s001.zip › Supplementary Files S3.pdf]

RECORD Statement—Completed Extension Checklist

Manuscript: *Clinical and Multimorbidity Profile of Patients with Diabetic Peripheral Neuropathy According to Documented GLP-1–Based Medication Status: An Exploratory Single-Center Cross-Sectional Study*

Version assessed: revised manuscript, 18 pages. This file reports the RECORD extensions; the corresponding STROBE items are documented in the separate completed STROBE checklist.

RECORD item	Reporting recommendation	Location in the manuscript
RECORD 1.1	Specify the type of routinely collected data and, when possible, name the databases.	Abstract, p. 2, identifies medication-level records and active-patient exports from the Clinical County Emergency Hospital Bihor. Section 2.1, pp. 3–4, describes the DPN and condition-specific hospital exports.
RECORD 1.2	Report the geographic region and study timeframe in the title or abstract, when applicable.	Hospital name is reported in the Abstract, p. 2. The full location and timeframe are reported in Section 2.1, pp. 3–4: Oradea, Romania; February 2024–February 2026; extraction 15–30 July 2026. The clinical timeframe is not stated in the current Abstract.
RECORD 1.3	State database linkage in the title or abstract when linkage was conducted.	The Abstract, p. 2, states that medication-level records were aggregated by PatID. Person-level linkage across the condition-specific exports is described in Sections 2.1–2.2, pp. 3–4, but is not explicitly stated in the current title or Abstract.
RECORD 6.1	Describe population-selection codes or algorithms in detail, or explain why this is not possible.	Sections 2.1–2.2, pp. 3–4. Eligibility was membership in the export filtered as "Complicatii: Neuropatie periferica" with a valid PatID. The source files did not preserve the underlying ICD-code algorithm or clinical criteria.
RECORD 6.2	Reference validation studies of selection codes/algorithms or report study-specific validation.	Sections 2.1–2.2, pp. 3–4; Discussion, p. 14. No preserved coding algorithm, specialist-adjudication field, confirmatory neurological testing, or study-specific validation was available; possible diagnostic misclassification is acknowledged.
RECORD 6.3	Use a flow diagram to describe linkage and the number of linked individuals at each stage.	Section 3.1, p. 6; Figure 1, p. 11. The flow shows 7660 medication-level rows, aggregation to 4120 unique PatID values, and classification into 852 patients with and 3268 without a documented GLP-1-based medication.
RECORD 7.1	Provide codes and algorithms used to classify exposures, outcomes, confounders, and modifiers, or explain why they cannot be reported.	Sections 2.1–2.4, pp. 3–5. Conditions were classified by PatID membership in the corresponding exports; preserved ICD codes were unavailable. Medication mapping is described in Section 2.3, pp. 4–5, and supplied in Supplementary Table S1.
RECORD 12.1	Describe investigators' access to the database population used to construct the study population.	Sections 2.1–2.4, pp. 3–5, describe the supplied active-patient exports used for analysis. The investigators analyzed the available export files; direct access to underlying source records, patient-level timestamps, and diagnostic algorithms was not available.
RECORD 12.2	Describe data-cleaning methods.	Sections 2.2–2.4, pp. 4–5; Section 3.5, p. 12. Repeated rows were collapsed by PatID; brand names were mapped to active ingredients; missingness and unclassifiable entries were retained and reported; height and BMI plausibility checks were applied without manual correction.
RECORD 12.3	State the level and method of linkage and how linkage quality was evaluated.	Section 2.2, p. 4; Sections 3.1 and 3.5, pp. 6 and 12; Figure 1, p. 11. Person-level deterministic linkage used PatID. All 7660 medication rows contained PatID, no duplicate patient identifiers remained after aggregation, and repeated rows did not contain conflicting demographic or clinical values used in the analysis.
RECORD 13.1	Describe selection of included persons, including filters for data quality, availability, and linkage.	Section 2.2, p. 4; Section 3.1, p. 6; Section 3.5, p. 12; Figure 1, p. 11. The complete active-patient DPN export with valid PatID was used. Available-case and complete-case denominators are reported for incomplete variables and regression models.

RECORD item	Reporting recommendation	Location in the manuscript
RECORD 19.1	Discuss implications of data not originally collected for the research question, including misclassification, unmeasured confounding, missing data, and changing eligibility.	Discussion, pp. 13–14. The manuscript addresses export-defined diagnoses, active-patient selection, lack of temporal information, exposure misclassification, missing anthropometric and laboratory data, residual confounding, and inability to determine treatment indication or outcome timing.
RECORD 22.1	Explain access to protocol, raw data, and programming code.	Data Availability Statement, p. 15. Patient-level records cannot be shared publicly because of ethical and institutional restrictions. Aggregate results are reported, and the medication-mapping dictionary, analysis code, and completed reporting checklists are identified as supplementary or controlled-access materials.

Checklist source: RECORD Statement checklist: https://www.record-statement.org/checklist.php
